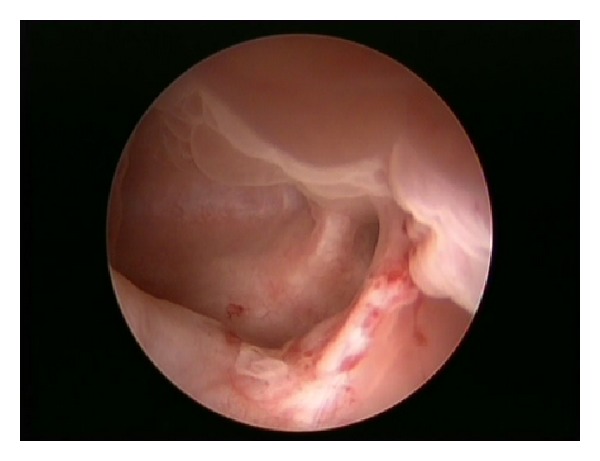# Urethral Diverticulum Calculi in a Male: A Case Report

**DOI:** 10.1155/2013/437106

**Published:** 2013-01-16

**Authors:** Emil Gadimaliyev

**Affiliations:** Department of Surgery, Ealing Hospital NHS Trust, Uxbridge Road, London UB1 3HW, UK

## Abstract

A 42-year-old male presented to the urology department, complaining of frequency and dysuria. A large number of calculi were revealed on IVU and USS. On endoscopic investigation, there were 3 stones (1.5 × 1 cm) found in the bladder and 5 more (1.5 × 0.8 cm) in the diverticulum of the posterior urethra. All of the stones were successfully broken down via a transurethral approach. This paper contains a detailed description of the case.

## 1. Introduction

A 42-year-old male presented to the urological department, complaining of frequent, painful, and difficult urination, which initially started a few years before. An occasional passage of small calculi was also noted to have taken place with urination. Two triangular shaped stones (1 × 0.8 cm in size) were reported to have passed during a period of an acute urinary retention a month before. A repeated acute urinary retention initiated the patient to approach a urologist. A total of 8 stones were revealed on IVU and the ultrasound study of the urinary tract. Stones, which were found on plain urography, were identical to the ones passed a month before, in terms of their colour and shape. The patient was offered a cystoscopy with an attempt of a transurethral stone extraction.

## 2. Case Presentation

A 12° optic 25 Fr cystoscope was passed in under spinal anaesthesia. The bladder was normal with no pathology identified. Urethra cystoscopy only revealed 3 triangular shaped, grey colored stones, whereas the IVU during the case visualised 8 well-defined shadows indicative of calculi. 

After removing the urethra cystoscope, a 26 Fr scope was inserted with a special ultrasonic lithotripsy probe for bladder stones. With the use of an ultrasound contact lithoclast, all 3 bladder stones were broken down and washed out. 

Further examination of the bladder neck revealed extra foramen on the posterior urethra at 7-8 o' clock position. An insertion of a nephroscope to this opening revealed a cavity with a mucosal looking inner layer, which contained 5 grey coloured, triangular shaped stones. 

With the use of a grasping forceps, the stones were relocated to the bladder, where they were successfully broken down and removed. The diverticulum was inspected following the clear out. It was found to be multichambered and extending directly under the bladder triangle. The prostate and the posterior urethra were found to be normal. 

The operation lasted a total of 90 minutes. After a successful trial without a catheter, the patient was discharged home the following day. 

**Figure 1:**
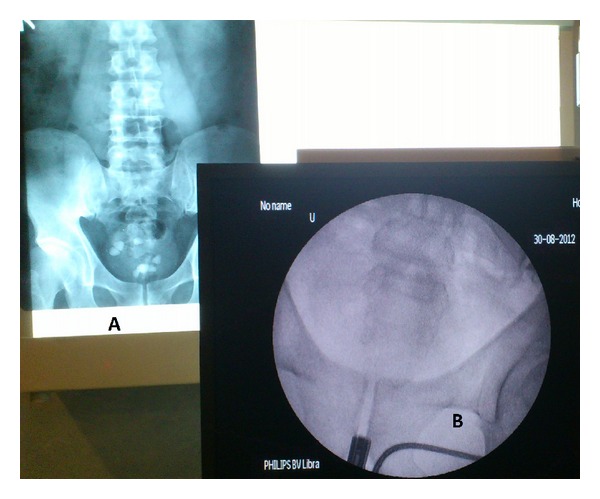


**Figure 2:**
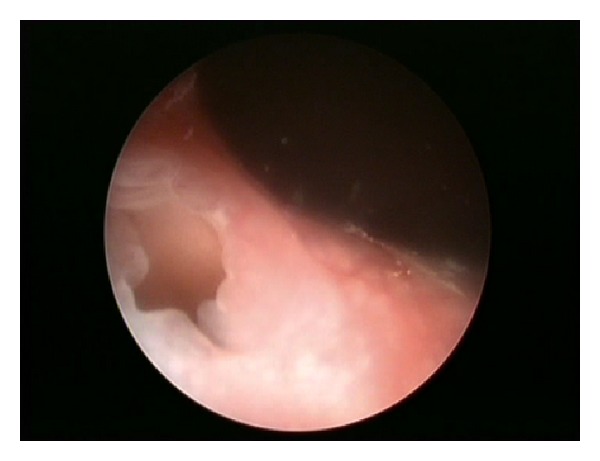


**Figure 3:**
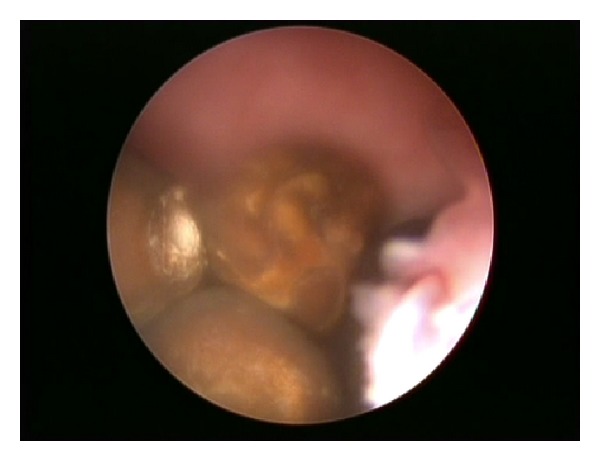


Below are some of the illustrations recorded intra-operatively Figures 1, 2, 3 and 4. 

## 3. Discussion

Congenital diverticulum of the urethra is a rarely seen anomaly [1, 2]. It presents as a sack-form enlargement of the back wall of the urethra—the body, communicating by a narrow track—the neck. These diverticula usually have the same layers as the urethra and outpouch the mucosal membranes from inside. They are often divided into two types—saccular or tubular, with the saccular type more associated with urinary obstruction and the tubular type more so with urinary stasis and calculosis [3]. 

Congenital urethral diverticula are mainly located at the level of the anterior urethra, often associated to anterior valves and can be found on the ventral side of the urethra [4]. The size of the UD may range between 1 and 6 cm in diameter. There are reported cases of UD arising from behind the glans of penis, stretching backward till the urethral bulb, causing a deformation of the penis and scrotum [5]. 

Congenital UD formation may be explained by an abnormal development in the area of ectodermal closing the urethral groove [6]. Since the diverticulum wall contains no muscular layer, there is no cavernous tissue around it. The wall tends to dilate and form a very thin walled sac filled with urine. It can only be emptied by means of an external pressure. Absent contractility of the diverticulum wall creates favourable conditions for urine stagnation and subsequent infection of its contents and calculus formation. 

 In our paper, we described a case of a large UD of the posterior wall in a male. This diverticulum was most likely congenital in origin, since no history of urinary infection, trauma, or catheterisation was reported. 

We found 12 other identical published cases concerning posterior urethral diverticula [7–9]. Since the posterior wall UD is a rare condition in males, there are no guidelines on operative treatment techniques. We propose that an endoscopic dilatation of the neck of the diverticulum with stone extraction and followup may be an alternative to the operative extraction of them, which may necessitate a perineal approach.

## Figures and Tables

**Figure 4:**